# A novel tool for evaluating children's musical abilities across age and culture

**DOI:** 10.3389/fnsys.2013.00030

**Published:** 2013-07-10

**Authors:** Isabelle Peretz, Nathalie Gosselin, Yun Nan, Emilie Caron-Caplette, Sandra E. Trehub, Renée Béland

**Affiliations:** ^1^Department of Psychology, International Laboratory of Brain, Music, and Sound Research, University of MontrealMontreal, QC, Canada; ^2^National Key Laboratory of Cognitive Neuroscience and Learning, School of Brain and Cognitive Sciences, Beijing Normal UniversityBeijing, China

**Keywords:** evaluation, musical abilities, children, amusia, tone language

## Abstract

The present study introduces a novel tool for assessing musical abilities in children: The Montreal Battery of Evaluation of Musical Abilities (MBEMA). The battery, which comprises tests of memory, scale, contour, interval, and rhythm, was administered to 245 children in Montreal and 91 in Beijing (Experiment 1), and an abbreviated version was administered to an additional 85 children in Montreal (in less than 20 min; Experiment 2). All children were 6–8 years of age. Their performance indicated that both versions of the MBEMA are sensitive to individual differences and to musical training. The sensitivity of the tests extends to Mandarin-speaking children despite the fact that they show enhanced performance relative to French-speaking children. Because this Chinese advantage is not limited to musical pitch but extends to rhythm and memory, it is unlikely that it results from early exposure to a tonal language. In both cultures and versions of the tests, amount of musical practice predicts performance. Thus, the MBEMA can serve as an objective, short and up-to-date test of musical abilities in a variety of situations, from the identification of children with musical difficulties to the assessment of the effects of musical training in typically developing children of different cultures.

## Introduction

Over the past several years, there has been considerable progress in identifying and describing disorders of music processing that arise from brain damage (e.g., Peretz et al., 1994) or are independent of such damage (i.e., “congenital amusia” Peretz, 2001). In the latter case, early identification of musical difficulties is desirable. Indeed, individuals with congenital amusia are unable to recognize well-known tunes in the absence of lyrics, and they have difficulty differentiating melodies on the basis of pitch cues alone, despite having normal hearing, speech, and intellectual ability, and ample opportunity for musical exposure (for reviews, see Peretz, 2008; Stewart, 2011). The common assumption is that these amusic individuals have not experienced normal musical development, but there has been little exploration of their music processing skills in childhood. The primary goal of the present study was to provide a means of identifying musical disorders in childhood, with the long-range goal of illuminating the course of abnormal musical development and its consequences for non-musical domains.

From a theoretical perspective, amusia offers a unique opportunity for examining the biological basis of music by tracing causal links among genes, brain, and behavior (Peretz, 2008). There is accumulating evidence that congenital amusia is hereditary (Drayna et al., 2001; Peretz et al., 2007) and is associated with reduced neural connectivity between the auditory cortex and the inferior frontal gyrus on the right side of the mature brain (Hyde et al., 2006, 2007, 2011; Loui et al., 2009). The neurogenetic origin of congenital amusia implies that vulnerability for this disorder can be present at birth. To date, this condition is mainly diagnosed with behavioral responses obtained on a battery of tests—the Montreal Battery of Evaluation of Amusia, or MBEA (Peretz et al., 2003). This battery has been validated for adults only.

Detection of amusia in childhood is clinically important because of the greater malleability of developing brains (Huttenlocher, 2002), offering the possibility of early intervention to ameliorate or compensate for such difficulties. The potential benefits of such intervention are substantial. Musical activities seem to shape cortical as well as subcortical neural structures (Kraus and Chandrasekaran, 2010), with beneficial consequences for intelligence and academic performance (Schellenberg, 2004, 2011), executive functions (e.g., Palleson et al., 2010), speech perception (e.g., Strait et al., 2011) and literacy (e.g., Moreno et al., 2009). Although little is known about the mechanisms that mediate this transfer of training (Peretz, 2008), the provision of early music training seems like a prudent course of action, as does tracking progressive changes in musical and non-musical abilities.

At present, detection of amusia can only be achieved after 10 years of age. At that age, children can complete the MBEA, which is, as mentioned, the most widely used tool for the evaluation of musical disorders in adults (Stewart et al., 2006). The MBEA consists of six tests that assess different components of Western tonal music, including contour, key (or scale), intervals, rhythm, meter, and memory. Individuals whose global score (averaged across the six tests) is two standard deviations below the mean of normal controls are considered amusic. By this statistical criterion, amusia affects 2.5% of the general adult population. If we only consider the scale test of the MBEA, which is the most diagnostic test of amusia and requires participants to discriminate pairs of melodies that may differ by a single tone that is out-of-key, the prevalence of amusia is 3.2% (the percentage of test takers who perform below the cut-off of 22 out of 30 correct responses). According to the same survey that includes more than 1000 university students (mean age: 24 years), the prevalence goes down to 1.5% of the population if we also consider those participants who fail to detect an out-of-key note in the same melodies (Provost, 2011). Accordingly, we may assume that genuine musical pitch deficits occur in at least 1% of the children.

Indeed, amusia can be observed in childhood. We had the opportunity to study a 10-year-old girl who was referred for persistent singing difficulties by her choir director (Lebrun et al., 2012). Her disorder was diagnosed with the abbreviated version of the Montreal Battery of Evaluation of Musical Abilities (MBEMA) presented here in Experiment 2. She exhibited deficits in all musical abilities that were assessed: melody, rhythm and memory. Her profile differed from the typical profile of amusic adults, who consistently fail on melodic discrimination tasks but do so less consistently on rhythmic tasks (Peretz, 2001; Hyde et al., 2006; Nan et al., 2010). However, at a slightly older age (10–13 years), it seems that the adult profile of amusia can be observed (Mignault-Goulet et al., 2012). Like their adult counterparts, these amusic pre-adolescents exhibited uniform difficulty in melodic processing but lesser difficulty in rhythm processing. Furthermore, all had difficulty detecting out-of-key notes in melodies, which, as mentioned above, is the hallmark of congenital amusia (e.g., Peretz et al., 2007). These findings raise the possibility that melodic difficulties persist across the lifespan while rhythmic problems may resolve over time. The study of younger children may address this issue while also elucidating the behavioral markers of amusia in early childhood.

The ensuing question is how early amusia can be diagnosed with behavioral measures. The available literature suggests that the processing components of the MBEA—melodic contour, intervals, keys, rhythm, meter and memory—are functional by 6 years of age (Trehub and Hannon, 2006; Hannon and Trainor, 2007; Stalinski and Schellenberg, 2012). Well before their first birthday, infants discriminate changes in pitch direction or melodic contour (Chang and Trehub, 1977; Trehub et al., 1987), and they detect subtle interval changes (i.e., pitch changes that preserve melodic contour) when the melodies are tonal (Cohen et al., 1987). In contrast, the differentiation of in-key from out-of-key changes in tonal melodies is not apparent until 4 or 5 years of age (Trainor and Trehub, 1994; Corrigall and Trainor, 2010). Infants also differentiate contrasting musical rhythms and meters (Hannon and Trehub, 2005; Hannon and Trainor, 2007), and they remember melodies for days or weeks (Saffran et al., 2000; Trainor et al., 2004; Volkova et al., 2006). By 6 years of age, then, one would expect typically developing children to be capable of completing the component tests of the MBEA with suitable adjustments for age and attentional capacity.

Because the stimuli from the MBEA are non-verbal, the tests are applicable, in principle, to listeners from a variety of language and cultural backgrounds. Children who speak a tone language are of particular interest because their early exposure to lexical tones could fine-tune their pitch discrimination abilities, with positive transfer to musical pitch processing (Wong et al., 2012). Deutsch et al. (2006) have suggested that early exposure to a tone language enhances pitch memory and the likelihood of attaining absolute pitch (i.e., memory for exact pitch levels). Others have suggested tone language facilitation for pitch perception and imitation (Pfordresher and Brown, 2009, but see Peretz et al., 2011, for a contrasting view). A critical question is whether tone language experience immunizes individuals against disorders of musical pitch processing. It does not (Jiang et al., 2010; Nan et al., 2010). About 3% of Chinese adults tested with the MBEA exhibit the typical profile of Western amusia, and differences between Mandarin speakers and French speakers are very small (Nan et al., 2010). It remains to be determined whether speakers of tone and non-tone languages would exhibit similar or divergent profiles of performance in childhood.

Music training also has the potential to moderate the expression of musical disorders in childhood. Music lessons have demonstrable effects that go well beyond improved discrimination of melody and rhythm (Forgeard et al., 2008). Hyde et al. (2009) found, for example that 6-year-old children who received 15 months of music lessons exhibited changes in motor and auditory regions of the cortex that correlated with performance on a variety of auditory and motor tasks. Above and beyond maturation, music training seems to have large and long-lasting effects, especially when training occurs early in development (Bailey and Penhune, 2012). Thus, one would expect early music lessons to result in improved performance on the MBEMA for typically and atypically developing children.

The major goal of the present investigation was to provide a means of identifying amusia in young children from different language and cultural backgrounds. In Experiment 1 a modified MBEA battery—the MBEMA—is used to assess a large sample of children from Canada and China. A more convenient version of the MBEMA (<20 min) is used with Canadian children (and young adults) in Experiment 2.

## Experiment 1: the montreal battery for evaluation of musical abilities (MBEMA)

The battery for adults (MBEA) consists of six tests (180 test items) that use a common pool of 30 novel musical phrases that are consistent with Western tonality and of sufficient complexity to guarantee meaningful musical processing. Furthermore, the MBEA is theoretically motivated and meets conventional psychometric criteria. It is sensitive, with normally distributed scores, good test-retest reliability, and scores that are correlated with those obtained on Gordon's *Musical Aptitude Profile* (Peretz et al., 2003). In our experience, the MBEA cannot be used with children younger than 10 years of age.

Adapting the MBEA for younger children is relatively straightforward because the task demands are minimal. Listeners are required to judge whether two successive melodies are the same or different on tests of scale (key), contour, interval, and rhythm. In addition, when queried on the memory test whether or not melodies are heard previously, they simply answer *yes* or *no*. In contrast, the length of the MBEA is excessive for children, taking well over an hour to complete. One way of making the MBEA more child-friendly is to reduce the length of the melodies (from 10 notes to 7, on average), the number of test items (from 30 to 20), and to eliminate the metric test, which proves too difficult for 6-year-olds in pilot testing.

### The test battery

**Figure 1:**
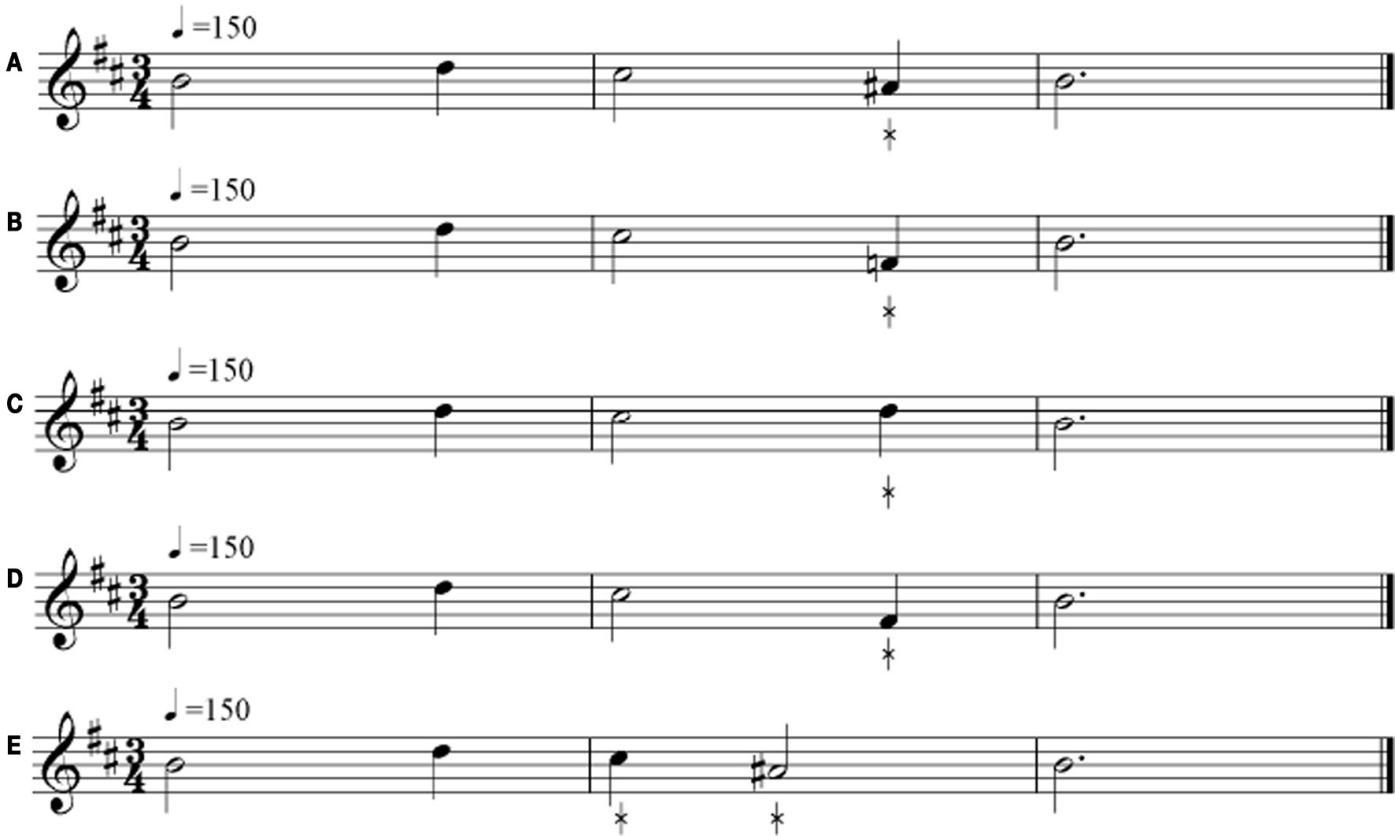
**Example of one musical stimulus as used in the five tests of the MBEMA**. The standard stimulus is represented in **(A)**, its scale alternate in **(B)**, its contour alternate in **(C)**, its interval alternate in **(D)**, and its rhythm alternate in **(E)**. The asterisk indicates the changed note. The example can be heard at www.brams.umontreal.ca/short/mbea-child

The MBEMA contains contour, interval, scale, rhythm, and memory tests. All tests use the same 20 unfamiliar tonal melodies, in 10 different keys (half major, half minor). As noted, the melodies are shorter versions of the musical phrases used in the (adult) MBEA (see Figure 1 for one example). The melodies, which are 5–9 tones long (M = 7.1) and 3–4 s (M = 3.5 s) in overall duration, are computer-generated, each presented in a different timbre or instrument. Ten different timbres (e.g., piano, marimba, guitar, flute) are used to make the tests as engaging as possible.

In the *melodic organization tests*, three types of manipulations are applied to the same 10 melodies (Figure 1). Comparison melodies that are different in the *scale* test introduce one out-of-key note while retaining the original melodic contour (i.e., a scale-violating change). Comparison melodies that are different in the *contour* test have one changed note that alters the pitch direction of the surrounding intervals while maintaining the original key (i.e., a contour-violating change). Comparison melodies that are different in the *interval* test have one changed note that alters intervals while preserving the original contour and key (i.e., an interval-violating change). The serial position of the modified note varies across melodies and never occurs in the first and last position. Average pitch interval changes are equivalent across the three conditions, with a mean of 3.6 (range: 2–5), 3.7 (2–5), and 3.6 (2–5) semitones from the original pitch for *scale*-violating, *contour*-violating and *interval*-violating changes, respectively.

Three sets of stimuli, each comprising 2 practice trials and 20 test trials, are constructed from these melodies. Each trial, which is preceded by a warning tone followed by 500 ms, consists of a target melody and comparison melody separated by a 1.5-s silent interval; trials are separated by 4-s silent intervals. The listener's task is to judge, on each trial, whether the target and comparison sequence are the same or different. The *scale* test, presented first, has 10 trials with identical (same) comparison melodies and 10 trials with scale-violating (different) comparison melodies. The *contour* and *interval* tests are similar to the *scale* test in using the same target melodies; they differ only in having *contour-violating* or *interval-violating* changes, respectively, in their comparison melodies. Melody pairs are always presented with the same timbre but in random order in each test.

The *rhythm* test uses the same melodies as the melodic organization tests. Here, the manipulation consists of changing the durations of two adjacent tones so as to alter the rhythmic grouping of notes while retaining the number of notes and original meter. This is accomplished by changing two quarter notes to a dotted quarter and an eighth note, or by reversing the order of two successive duration values (e.g., a half note followed by a quarter note becomes a quarter note followed by a half note, as illustrated in Figure 1). The serial position of these changes varies across melodies. A set of 2 practice trials and 20 test trials is constructed with these stimuli. This task also requires a same-different response.

The final test of the music battery assesses recognition *memory*. From the initial set of 20 melodies, 10 are selected for the recognition phase. Each is presented previously at least four times in the same format. In addition to these old melodies, there are 10 foils or new melodies. The 10 new melodies are constructed in the same manner as the old melodies but differ in their exact temporal and pitch patterns. The 20 sequences are then recorded in random order with 4-s inter-trial intervals for response. Participants are required to respond *yes* if they recognize a melody from earlier in the session and to respond *no* otherwise. This test assesses incidental memory because children are not informed that their memory for the melodies will be tested subsequently.

### Method

#### Participants

The Canadian sample consisted of 245 children 6–8 years of age from public and private schools in the Montreal area. Exclusion criteria include brain trauma, hearing deficits, attentional deficits and learning disabilities (e.g., dyslexia) as reported by the parent. The data of 13 additional children were discarded from the analyses because information regarding musical training or birth date was missing or because they spoke a tonal language at home. The characteristics of the studied samples are summarized in Table 1. Parents, who provided informed consent, reported that most children were right-handed (88.2%), French-speaking (76.3%), and had no private music lessons (73%). The Chinese sample consisted of 91 same-age children from Beijing. All were native speakers of Mandarin, a tone language. Their demographic characteristics are also summarized in Table 1. As can be seen, a majority of Chinese children (72.5%) had private musical lessons and much more extracurricular music education than their Canadian counterparts, *t*_(334)_ = 9.50, *p* = 0.001.

**Table 1:** **Characteristics of the children tested on the full MBEMA in Experiment 1**.

**Age**	**6**	**7**	**8**	**Total**
**CANADIAN CHILDREN**				
Sample size (Gender)	67 (39M, 28F)	92 (40M, 52F)	86 (31M, 55F)	245
School grade				
Kindergarten	17	–	–	17
Grade 1	50	37	–	87
Grade 2	–	55	66	121
Grade 3	–	–	20	20
Music training				
No lesson	53	74	53	180
Lessons (mean duration in months)	14 (13.6)	18 (13.6)	33 (20.5)	65 (17.1)
From age 4	2	2	1	5
From age 5	5	0	3	8
From Age 6	7	7	9	23
From age 7	–	9	11	20
From age 8	–	–	9	9
**CHINESE CHILDREN**				
Sample size (gender)	29 (15M, 14F)	30 (14M, 16F)	32 (17M, 15F)	91
School grade				
Kindergarten	–	–	–	–
Grade 1	29	7	–	36
Grade 2	–	23	13	36
Grade 3	–	–	19	19
Music training				
No lesson	8	9	8	25
Lessons (mean duration in months)	21 (20.5)	21 (22.4)	24 (35.9)	66 (25.7)
From age 2	2	–	–	2
From age 3	1	2	4	7
From age 4	9	3	7	19
From age 5	5	6	2	13
From age 6	4	8	7	19
From age 7	–	2	4	6

#### Procedure

The five tests were presented to each child individually in a quiet room in their school in a session that lasted 30–45 min. The order of presentation was fixed, with scale, contour and interval tests followed by the rhythm and memory tests. The tests were presented through computer loudspeakers at a comfortable listening level, and the children responded verbally. They were free to request breaks between tests, and each child received a token gift for participation.

### Results and discussion

Results from the Canadian sample are presented for sensitivity, age, music lessons, and amusia, followed by an examination of the Chinese results and Canadian–Chinese comparisons. Preliminary analyses revealed that gender did not influence performance in the Canadian sample. In the Chinese sample, girls performed better than boys, *F*_(1, 89)_ = 8.06, *MSE* = 57.71, *p* = 0.006. Because gender did not interact with any other factor, gender was not considered further.

#### Sensitivity

**Figure 2:**
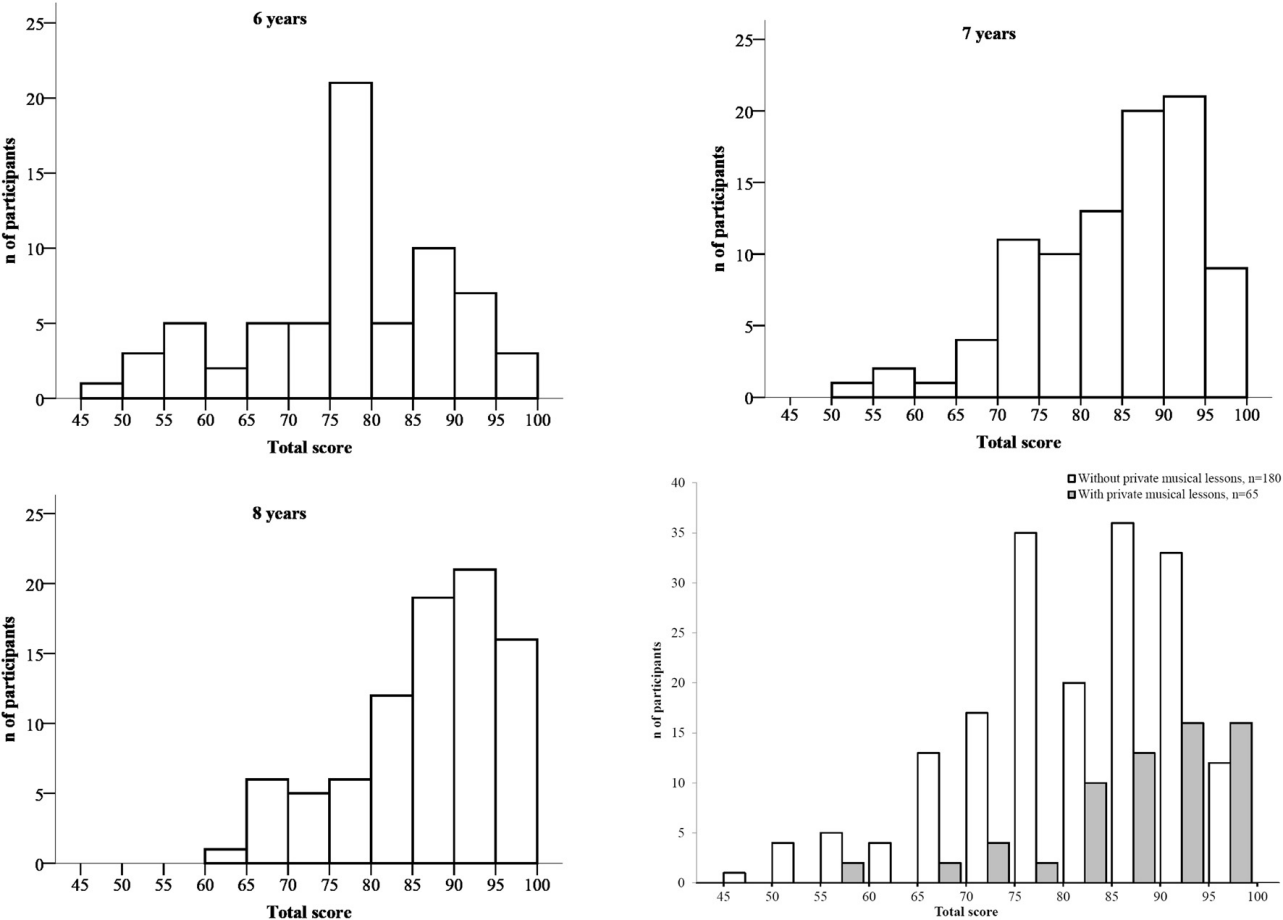
**Distribution of the total scores as a function of age on the full MBEMA in Canadian children (*N* = 245)**. The distribution of children without private music lessons and of the children with music lessons is presented in the lower right panel.

Canadian children's global scores (across the five tests) at 6, 7, and 8 years of age ranged from 47 to 90 with 50 representing chance performance and 100, a perfect score. As can be seen in Figure 2, the distribution of cumulative scores was positively skewed but did not violate normality [*D*_(67)_ = 0.99, *p* = 0.282; *D*_(92)_ = 1.09, *p* = 0.18; *D*_(86)_ = 1.28, *p* = 0.075, for 6-, 7-, and 8-year-olds, respectively, by Kolmogorov–Smirnov test]. However, scores on individual tests were positively skewed, violating normality in most cases. Thus, the total score is more sensitive than individual test scores in distinguishing normal from abnormal performance. Indeed, if the distribution of scores is not normal, but negatively skewed, the proportion of the general population estimated to be afflicted with congenital amusia will increase (Henri and McAuley, 2010).

#### Age

**Figure 3:**
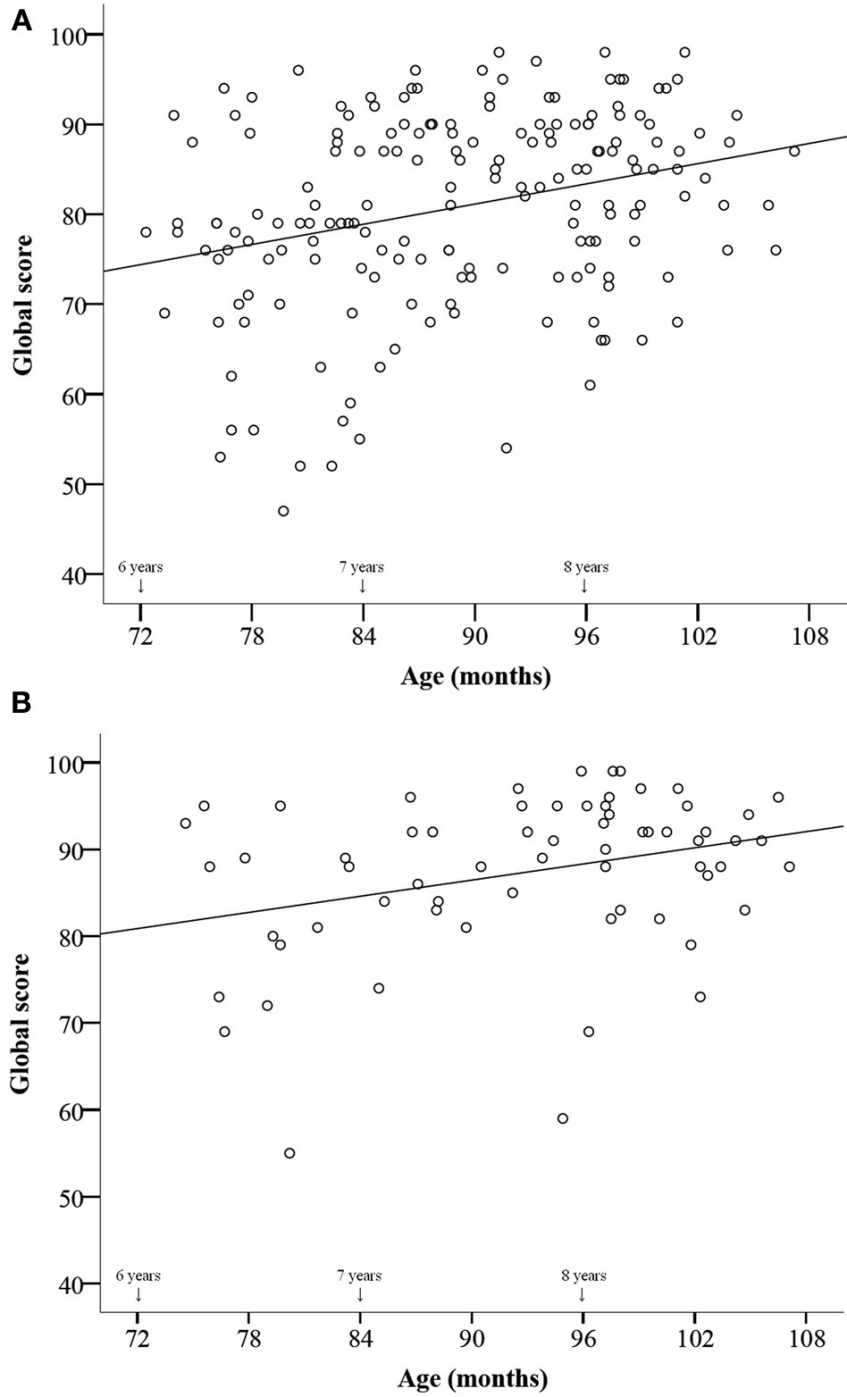
**Plot of individual total scores on the full MBEMA as a function of age in Canadian children who have not received private music lessons (A), and who have had music lessons (B)**. There was a significant effect of age in each group, with *r*_(178)_ = 0.29, *p* = 0.001 for the untrained and *r*_(63)_ = 0.31, *p* = 0.012, for the musically trained children.

We measured age in terms of months and correlated it with the total scores (Figure 3) and the scores obtained on each test. Age predicted all scores, from global performance [*r*_(243)_ = 0.33, *p* = 0.001] to each test performance [*r*_(243)_ = 0.24, 0.28, 0.28, 0.22, 0.28 for the *scale, contour, interval, rhythm*, and *memory* test, respectively; all *p* < 0.002].

#### Music lessons

Private music lessons also mattered. Months of music lessons predicted total performance at 7 and 8 years, with *r*_(90)_ = 0.21, *p* = 0.046 and *r*_(84)_ = 0.27, *p* = 0.011, respectively, whereas at 6 years the correlation did not reach significance, *r*_(65)_ = 0.22, *p* = 0.072. This likely reflects the limited cumulative practice of 6-year-olds.

We also examined the role of musical training as a dichotomous variable as typically used in the literature, that is, by comparing performance between the 65 children with private musical lessons and the 180 children with no lessons (see Figure 2). To do so, we examined the effect of music training on performance by considering months of age as a covariate. Indeed, opportunities for music training increase with age. The ANCOVA considering musical lessons (with, without) as a between-subjects factor and test (scale, contour, interval, rhythm, memory) as a within-subject factor revealed a main effect of musical training, *F*_(1, 243)_ = 18.56, *MSE* = 21.84, *p* = 0.001. Music lessons provided an overall gain of 6% in performance on the MBEMA, irrespective of the test considered. The interaction between musical training and test was not significant [*F*_(4, 972)_ = 1.38, *MSE* = 3.39, *p* = 0.24].

#### Diagnosis of amusia

Congenital amusia is typically diagnosed in adults by a global score of 2 SD below the mean. Using the same criterion (see cut-off scores in Table 2), 11 children (3 at age 6, 4 at age 7, and 4 at age 8; one child in each age group has extracurricular music lessons) out of the 245 tested would be considered amusic. By this statistical criterion, however, the cut-off score for the musically-untrained 6-year-olds is 51.5, which is close to the chance level of 50 and may not be sensitive enough to the presence of a deficit. Nevertheless, children's difficulties do not seem limited to the melodic dimension but to involve rhythm as well. Note, however that the scores obtained on the MBEMA are highly variable at these ages.

**Table 2:** **Mean (SD) score on each test and on all tests (total) of the MBEMA along with the cut-off score corresponding to 2 SD below the mean, for Canadian and Chinese children in each age group**.

	***n***	**Scale (/20)**	**Contour (/20)**	**Interval (/20)**	**Rhythm (/20)**	**Memory (/20)**	**Total (/100)**	**Cut-off**	**Cut-off for musicians**	**Cut-off for non-musicians**
**CANADIAN**										
6 years	67	14.5 (2.9)	15.3 (3.4)	15.3 (2.8)	16.2 (2.7)	15.6 (3.6)	77.0 (12.2)	52.7	58.8 (*n* = 14)	51.5 (*n* = 50)
7 years	92	15.9 (2.4)	16.8 (2.6)	16.4 (2.7)	17.1 (2.6)	17.4 (2.4)	83.5 (9.8)	63.9	68.5 (*n* = 18)	63.0 (*n* = 74)
8 years	86	16.1 (2.3)	17.2 (2.2)	17.2 (2.1)	17.6 (2.3)	17.8 (2.5)	86.0 (9.0)	67.9	75.6 (*n* = 33)	65.1 (*n* = 53)
**CHINESE**										
6 years	29	16.3 (2.2)	17.9 (1.3)	16.2 (2.2)	14.7 (2.8)	16.0 (4.1)	81.1 (8.4)	64.4	63.8 (*n* = 21)	66.9 (*n* = 8)
7 years	30	15.6 (2.1)	17.3 (1.9)	17.7 (2.1)	17.2 (2.1)	17.8 (1.9)	85.6 (7.2)	71.2	76.4 (*n* = 21)	66.6 (*n* = 9)
8 years	32	16.4 (1.7)	17.9 (1.9)	18.2 (1.7)	18.4 (1.4)	19.0 (0.9)	89.9 (5.6)	78.7	78.9 (*n* = 24)	78.5 (*n* = 8)

Musicians are those children who had private music lessons; the number of children is presented into parentheses.

#### Cross-cultural comparison: Canadian and Chinese children

The distribution of total scores obtained by the Chinese children did not violate normality [*D*_(29)_ = 0.69, *p* = 0.74; *D*_(30)_ = 0.72, *p* = 68; *D*_(32)_ = 0.67, *p* = 0.76, for 6-, 7-, and 8-year-olds, by Kolmogorov–Smirnov tests]. No child obtained a perfect score, as was the case for Canadian children. Thus, the total score can distinguish typical from atypical music processing.

Age, as measured in months, was correlated with the scores obtained by Chinese children on most tests, with *r*_(89)_ = 0.37, 0.54, 0.41 for total performance, interval, rhythm and memory scores, respectively, all *p* = 0.001. However, age did not predict scores on the scale and contour tests [*r*_(89)_ = 0.03 and 0.06]. This might be due to the fact that children were tested first on the scale and contour tests.

As can be seen in Table 2, Chinese children obtained higher scores than Canadian children. However, more Chinese children had extracurricular music training, as noted previously, and for a longer period. This was the case in each age group, *t*_(94)_ = 5.664, *p* = 0.001, *t*_(120)_ = 6.350, *p* = 0.001, and *t*_(116)_ = 5.745, *p* = 0.001, for 6-, 7-, and 8-year-olds, respectively. Moreover, duration of musical training predicted performance in Chinese children, *r*_(89)_ = 0.35, *p* = 0.001; *r*_(89)_ = 0.26, *p* = 0.012, *r*_(89)_ = 0.21, *p* = 0.047, *r*_(89)_ = 0.21, *p* = 0.045; and *r*_(89)_ = 0.26, *p* = 0.014, for scale, contour, interval, rhythm and memory test, respectively.

To measure the effect of culture while controlling for music training an ANCOVA with months of music lessons as a covariate was conducted on the individual test scores. Culture (Canadian, Chinese) was the between-subjects factor, and test (scale, contour, interval, rhythm and memory) was the within-subject factor. Separate ANCOVAs for each age group were performed to minimize the contribution of age. The analysis revealed a significant interaction between test and culture in 6- and 7-year-olds *F*_(4, 372)_ = 5.49, *MSE* = 4.72, *p* = 0.001, and *F*_(4, 476)_ = 3.01, *MSE* = 3.20, *p* = 0.018, respectively, and a marginally significant interaction in 8-year-olds, *F*_(4, 460)_ = 2.21, *MSE* = 2.25, *p* = 0.067. More specifically, at 6 years, Chinese children performed significantly better than Canadian children on the scale, contour and rhythm tests [*t*_(94)_ = 3.075, *p* = 0.003; *t*_(94)_ = 3.98, *p* = 0.001; *t*_(94)_ = 2.422, *p* = 0.017], but not on the interval, and memory tests [*t*_(94)_ = 1.466, *p* = 0.14 and *t*_(94)_ = 0.427, *p* = 0.67, respectively]. This pattern changed by 7 years of age. Chinese children perform marginally better than Canadian children on the interval test only [*t*_(120)_ = 2.499, *p* = 0.014]. At 8 years, Chinese children outperformed Canadian children on the memory test only [*t*_(116)_ = 2.750, *p* = 0.007]. Note that the Chinese superiority was not limited to the processing of pitch and hence may not be related to the use of a tone language. Finally, only 3 Chinese children can be considered as potentially amusic since they perform 2 SD below the mean (Table 2).

## Experiment 2: abbreviated MBEMA

Whereas the MBEMA, as described in Experiment 1, is suitable for identifying children with amusia or with relatively poor music perception skills, it is impractical for general use. For example, music educators may wish to distinguish between poor performance in young children arising from inadequate effort or from underlying difficulties in music pattern perception. An abbreviated version of the MBEMA could serve as a preliminary screening tool, with poor outcomes leading to more comprehensive assessment involving audiometry and the full battery. Accordingly, we sought to examine the utility of a short form of the test battery. The battery was simplified by collapsing the three melodic tests (scale, contour and interval) into a single *melody* test. This new melody test includes 10 melodies that are repeated for “same” trials, and four scale-violating comparisons, three contour-violating comparisons, and three interval-violating comparisons for the “different” trials. Thus, the abbreviated battery consisted of three tests (melody, rhythm and memory), each with 20 trials. The rhythm and memory tests are identical to the corresponding tests of the full MBEMA, as described in Experiment 1. In contrast to the full version, which requires 30–45 min to administer, the abbreviated form takes approximately 20 min. Its suitability and validity are examined here with a new group of Canadian children of the same age as those tested in Experiment 1.

### Method

There were 85 Montreal-area children who were tested with the abbreviated MBEMA and 45 children who were tested with both the abbreviated and the full MBEMA in separate sessions, 39–81 days apart. Twenty-three were tested, first, on the full battery and, later, on the abbreviated form; the other half was tested in reverse order. All children were recruited and tested according to the same criteria used in Experiment 1 (Table 3). Most children were right-handed (85%), French-speaking (92%), with no private music lessons (78%). We also tested a comparison group of 28 young adults (23 women, 5 men, 18–20 years) from a junior college in Montreal. Most college students were right-handed (93%), French-speaking (100%), and had musical training (57%), with lessons from 8 to 60 months.

**Table 3:** **Characteristics of the children tested on the abbreviated MBEMA in Experiment 2**.

**AGE**	**6**	**7**	**8**	**Total**
**ABBREVIATED VERSION ONLY**				
Sample size (gender)	27 (11M, 16F)	31 (11M, 20F)	27 (13M, 14F)	85
School grade				
Kindergarden	22	–	–	22
Grade 1	5	25	1	31
Grade 2	–	6	19	25
Grade 3	–	–	7	7
Music training				
No lessons	24	24	18	66
Lessons	3	7	9	19
From age 4	1	–	–	1
From age 5	1	2	–	3
From age 6	1	3	4	8
From age 7	–	2	5	7
**ABBREVIATED AND FULL VERSION**				
Sample size (gender)	16 (11M, 5F)	14 (5M, 9F)	15 (3M, 12F)	45
School grade				
Kindergarden	11	–	–	11
Grade 1	5	14	–	19
Grade 2	–	–	15	15
Music training				
No lessons	10	10	10	30
Lessons	6	4	5	15
From age 4	1	–	–	1
From age 5	4	3	–	7
From age 6	1	1	–	2
From age 7	–	–	4	4
From age 8	–	–	1	1

The apparatus and procedure were the same as in Experiment 1.

### Results and discussion

None of the 85 children obtained a perfect score, confirming the sensitivity of the abbreviated battery. Scores on the tests were mostly normally distributed [global score: *D*_(85)_ = 1.07, *p* = 0.19; melody: *D*_(85)_ = 1.28, *p* = 0.07; memory *D*_(85)_ = 0.1.14, *p* = 0.151], with the exception of the rhythm test [*D*_(85)_ = 1.55, *p* = 0.016, by Kolmogorov–Smirnov test]. The scores were very similar across age groups (Table 4).

**Table 4:** **Mean scores (and SD) and cut-off scores obtained by Canadians with the abbreviated MBEMA as a function of test and age**.

	***n***	**Melody/20**	**Rhythm/20**	**Memory/20**	**Global Score (% correct)**	**Cut-off (% correct)**
6 years	27	14.4 (1.8)	16.5 (2.3)	15.4 (2.1)	77.3 (8.4)	60.5
7 years	31	15.2 (3.0)	16.7 (2.9)	15.9 (2.5)	79.6 (10.6)	58.4
8 years	27	15.2 (2.2)	16.8 (2.1)	15.8 (1.9)	79.6 (6.6)	66.4
Adults	28	17.1 (2.0)	18.1 (1.5)	18.0 (1.4)	88.8 (6.3)	76.2

Age, measured in months, did not predict performance on any of the tests [melody: *r*_(83)_ = 0.19, *p* = 0.08; rhythm: *r*_(83)_ = 0.04, *p* = 0.74; memory: *r*_(83)_ = 0.13, *p* = 0.22; global score: *r*_(83)_ = 0.16, *p* = 0.15]. In contrast, music training had a noticeable effect, especially on the melody test, *r*_(83)_ = 0.21, *p* = 0.028, by one-tailed test.

Adult scores were generally high (Table 4), but no adult obtained a perfect score. Their scores were significantly higher than those of 8-year-old children, *F*_(1, 55)_ = 24.80, *MSE* = 5.14, *p* = 0.001, and there was no interaction between age and test, *F*_(2, 110)_ = 0.96, *MSE* = 2.72, *p* = 0.38. In order to take into account the fact that adults have more music training than 8-year-olds, *t*_(55)_ = 3.65, *p* < 0.005, an ANCOVA with music training as a covariate was conducted; this analysis revealed that the age effect was still significant, *F*_(1, 54)_ = 15.96, *MSE* = 5.12, *p* = 0.001.

Finally, of the 4 children who scored at or below 2 SD from the mean, none showed a melodic deficit, 3 had a rhythmic deficit, and 3 had a memory deficit. In short, the predominant melodic deficit found in adults was not evident with the abbreviated battery either.

#### Validation with the full battery

To allow comparison of the full and abbreviated MBEMA, the melodic scores obtained on the scale, contour, and interval test of the full MBEMA were averaged in a single score (maximum score is 20). When reduced to three (melody, rhythm and memory) scores, the full battery yielded slightly better performance (86% correct) than the short form (85.1%). The difference was more pronounced at the first test session, with children achieving 83.8% correct on the full MBEMA and 78.7% on the abbreviated form. Performance on the abbreviated form was slightly better when it occurred in the second test session. This pattern was confirmed by an interaction between test order and battery form, *F*_(1, 43)_ = 63.81, *MSE* = 25.15, *p* = 0.001. Furthermore, melodic and to some extent rhythmic scores correlated significantly between the two versions of the battery, *r*_(43)_ = 0.49, *p* = 0.001 and *r*_(43)_ = 0.27, *p* = 0.07, respectively. However, memory scores were uncorrelated across the two versions, *r*_(43)_ = 0.10, *p* = 0.49. Memory scores are generally higher with the full MBEMA at retest [17.7 vs. 18.4/20; *t*_(21)_ = 3.76, *p* = 0.001]. The scores may reflect different forms of memory. The memory test was initially presented as a test of incidental memory. At retest, it may function as an explicit memory test for children previously familiarized with the procedure. This possible carry-over effect should be taken in consideration when both versions of the MBEMA are used with the same children.

Note, however, that retest is advisable in the case of suspected difficulties because in this sample, we identify two 7-year-old children as presenting with an amusic score (one with each form of the battery). On re-test, performance fell in the (low) but normal range. Thus, test familiarization, especially as the child matures, plays an important role.

## Conclusions

Research on normal and impaired music processing has gained increased attention in the past decade alongside interest in the relationship between music and language (Patel, 2012) and interest in musical training as a framework for brain plasticity (Herholz and Zatorre, 2012). In this regard, assessment of musical abilities has become a central issue. However, evaluation of basic music processing components has so far been limited to adults, probably due to the lack of a reliable and valid tool for children. Here we present the Montreal Battery for Evaluation of Musical Abilities (MBEMA) that is suitable for assessing musical abilities and disorders in Western and Asian children 6–8 years of age. In effect, the full and abbreviated forms of the battery present neither floor nor ceiling effects, with global scores normally distributed around a mean of 82% for Canadians (Chinese: 85%) and 79% for the abbreviated version. Thus, the MBEMA shows better sensitivity than the Montreal Battery of Evaluation of Amusia (MBEA) from which it derives. With the MBEA, a musically untrained adult has a mean accuracy of 87% (range: 72–99). The battery is therefore likely to exhibit ceiling effects when used with musically trained individuals. In contrast, we find here that both versions of the MBEMA are suitable for identifying individual differences in music perception and memory in children from different musical and cultural backgrounds.

To facilitate future research, the two versions of the MBEMA, along with the norms, are freely accessible from our Web site www.brams.umontreal.ca/short/mbea-child (as is the case for the adult version of the MBEA). It is worth pointing out here that the MBEMA, in both versions, successfully distinguishes children with musical training from those who do not have private music lessons. Presumably, the scores reflect cumulative practice. As mentioned, no one reaches a perfect score, even when the abbreviated version is completed by young adults with 5 years of music lessons (Experiment 2). The abbreviated version takes less than 20 min to complete. Thus, the abbreviated MBEMA can serve as an objective, short, and up-to-date test of musical abilities in a variety of situations, from the identification of children with musical difficulties to the assessment of the effects of musical training in typically developing children and perhaps even adults.

Interestingly, Mandarin-speaking children score higher than their Canadian (French- and English-speaking) counterparts on the tests, even when extra-curricular musical education is held constant. Contrary to our previous study (Wong et al., 2012), the Chinese advantage is not limited to the tests that assess pitch-based processing but extends to rhythm discrimination and memory. This finding raises the possibility that the enhanced perceptual and cognitive processing for musical pitch that is often reported in tone language speakers (e.g., Bidelman et al., 2013) is not related to language background but to other cultural differences. There are cultural differences in worldview, representations of self, and even thinking styles: East Asians tend to be more collectivist, interdependent, and holistic, while Westerners tend to be more individualistic, independent, and analytic. These broad cultural differences have been used to explain differences in visual perception (Nisbett et al., 2001; Chua et al., 2005). Similarly, these cultural differences may account for music perception. Westerners may focus more on the local note change while East Asians may encode the melodies more holistically. According to the present results, the latter strategy would be more effective for completing the full version of the MBEMA (Experiment 1). Testing this hypothesis should be the goal of future studies.

Across cultures, special attention is necessary for children at risk of developing congenital amusia. Amusic children who speak a tone language may have the greatest need for habilitation. Amusic adults often have difficulty identifying (Nan et al., 2010) or discriminating lexical tones (Liu et al., 2012). Music training may ameliorate their processing difficulties to some extent, especially if the training is initiated in the early years. However, longitudinal research is necessary to confirm that children identified as amusic at 6–8 years of age continue to meet the diagnostic criteria some years later. If they do not, it would be important to ascertain whether intervention occurred in the intervening years. On the basis of the present findings, intervention should not be restricted to musical pitch processing. Melodic processing deficits are absent in a subset of amusic children, who exhibit greater difficulty with musical rhythm or memory. The pattern of deficits differs from the profile of amusic pre-adolescents (Mignault-Goulet et al., 2012) and adults (e.g., Peretz et al., 2003), who uniformly exhibit melodic processing deficits. Longitudinal studies would make it possible to ascertain whether there are different developmental trajectories for melody, rhythm, and memory.

Age-related improvements are observed with the full MBEMA but not with its abbreviated form (as far as the 6–8 year-olds are concerned). Age-related change is particularly marked between 6 and 7 years, corresponding to the first and second years of schooling, respectively (Table 2). Improved performance is unlikely to be attributable to formal schooling or gains in literacy because there is no evidence of abrupt change when finer-grained measures of age (months rather than years) are considered. It is likely that improvements in pitch resolution (e.g., Maxon and Hochberg, 1982), strategic listening (Eisenberg et al., 2000), and memory (Barouillet et al., 2009) contribute to the age effects. Because the full battery differs from the abbreviated form primarily in its greater demands on attention and motivation, these factors may account for age-related changes observed in performance on the full battery only.

To conclude, the critical advantage of a sensitive and valid tool for the diagnosis of amusia in childhood is its ability to guide intervention and to evaluate the efficacy of such intervention in the early years when the brain is most plastic. Above all, the battery can be used to distinguish true amusics from numerous cases of self-reported tone deafness with normal music processing abilities (Cuddy et al., 2005; Sloboda et al., 2005). Test results that confirm normal musical functioning can be used to alleviate anxiety and encourage musical engagement.

### Conflict of interest statement

The authors declare that the research was conducted in the absence of any commercial or financial relationships that could be construed as a potential conflict of interest.
